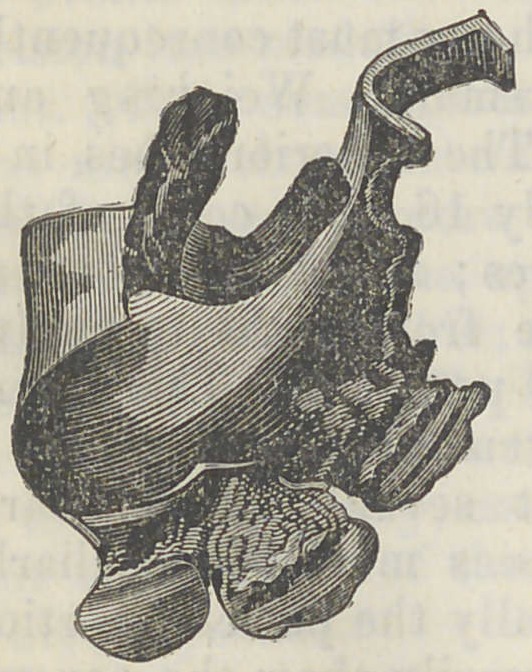# Cases in Country Practice

**Published:** 1862-12

**Authors:** John Ellis Blake

**Affiliations:** Middletown, Conn.


Cases in Country Practice.—*By John Ellis Blake, M. D., of Middletown, Conn.—No. VII.—Artificial Teeth Removed from the Œsophagus.*—In the early part of the present summer (1862,) I was called, in great haste, a short distance from town to see Mr. H——, a young man about 30 years of age, who (the messenger said) "was choking to death;" adding that he believed he had "*swallowed his teeth.*" Somewhat astonished at this, I took a few instruments with me, and was soon at the house. The young man was in a pitiable state, laboring for breath, his body bowed forward, his face livid, and once and a while clutching at his throat and mouth with his hands as if in extreme distress. It was now about 2 A. M., and it appeared that about an hour and a quarter before, whilst lying on his back, sound asleep, he had suddenly felt a suffocating sensation, and immediately after an involuntary effort at swallowing taking place, he found that his false teeth, of which he wore three ("front uppers") upon a plate, had gone down in his throat; and, what is worse, a moment afterwards an injudicious attempt to pull them up brought on another spasmodic attempt to swallow, which carried them far out of reach, and into a position where their pressure on the trachea caused extreme dyspnœa.

I give here a wood cut of the plate of the teeth, by which some idea of the difficulties to which its peculiar shape gave rise may be obtained. It will be seen that this is a plate of three teeth, bent upon itself at each side, and each curved portion fashioned into double prongs, so as to clasp about the natural teeth, and hold the set firmly in its place. The plate was of silver, and at the edges of all the prongs quite thin and dangerously sharp. The position of these prongs and edges are such, that in a flexible tube, like œsophagus an attempt to move it in either direction would be liable to cause a point to penetrate the tissues, and hold everything fast.

I found, after several futile attempts to withdraw it (in which I got a good hold upon it with a pair of very long, slender, curved forceps,) that it was firmly lodged in the œsophagus at a point between the top of the sternum and the thyroid cartillage. I presume it lay with its long axis (if I may so express it) across the tube, and so produced the greater dyspnœa. It evidently could only be removed by the use of considerable force; and putting aside the operation of removing it by incision from without as inadmissible, I proposed to try to get it *up* rather than to force it *down*, which, although it could be more easily done, would not be wholly free from danger, and promised by no means so satisfactory a result.

Keeping the blades of the long curved forceps before mentioned firmly closed, they were passed down by the side and below the plate until I could use them as a lever, then by a sort of prying and lifting process, turning the forceps strongly with the right hand, assisted by the pressure of the left hand on the neck, I forced the teeth up quite a distance. Although this was done as gently as was consistent with the force required, it was a cruel trial for the half suffocated patient. The tissues it seemed could almost be *heard* to tear, and I have no doubt, from the sensation conveyed through the instrument to my hand, and from the symptoms of the patient after the operation, that the œsophagus was somewhat lacerated. As no delay could be allowed in the passage of the teeth over the chink of the glottis, the forceps were laid aside, and as soon as the patient took breath, having a small hand, I was enabled to force the left one far enough into the fauces to engage the tip of the middle finger under the plate, and then by a quick pull to bring it out; to my own satisfaction, and I hardly need add to the great joy of the patient. Some small vessels, large enough to bleed smartly for a few moments, were opened; but the whole amount of hæmorrhage was trivial.

The operation, of which I have spoken so much at length, was accomplished in a few moments after I reached the house; but I think the case worthy of relation (although I do not know it to be an *isolated* one,) as a warning to those who having false teeth, are so careless to go to sleep without removing them from the mouth, or in some way making it certain to have them secure. A case, I remember, occurred in the practice of my perceptor and friend, Dr. J. Mason Warren, of Boston, in which, during etherization, a set of teeth fell back into the fauces, for a time threatening to suffocate the patient, and from which dangerous condition she was only rescued by extreme promptitude of action on the part of that surgeon.

I would remark here, that the natural, involuntary action of œsophagus, by which the food, after passing a certain point, is carried on towards the stomach, is a great source of embarrassment in cases like the one I have related; for the irritation created by the foreign body causes not only a constant spasmodic effort on the part of the patient to swallow, but puts the involuntary action in play, which is directly antagonistic to the efforts of the surgeon.

My patient suffered for some days from swelling and inflammation of the throat, and had great pain in swallowing, but was not long ill, and is now entirely well.—*Boston Medical and Surgical Journal.*

## Figures and Tables

**Figure f1:**